# Building community engagement with caregivers through online interaction and a salutogenic approach in a period of isolation

**DOI:** 10.3389/fmed.2024.1229395

**Published:** 2024-02-28

**Authors:** Olga Mas-Casadesús, Laura de la Torre-Pérez, Glòria Reig-Garcia, Anna Mas-Casadesús, Anna Berenguera, Dolors Juvinyà-Canal

**Affiliations:** ^1^Health and Health Care Research Group, University of Girona, Girona, Spain; ^2^Vielha e Mijaran Primary Health Centre, Aran Salut, Vielha e Mijaran, Spain; ^3^Primary Health Centre, CAPSBE (Consorci d’Atenció Primària de Salut Barcelona Esquerra), Barcelona, Spain; ^4^Methodology of Biomedical Research and Public Health PhD Program, Universitat Autónoma de Barcelona, Bellaterra, Spain; ^5^Department of Clinical Epidemiology and Public Health, Iberoamerican Cochrane Centre, Biomedical Research Institute Sant Pau (IIB Sant Pau), Barcelona, Spain; ^6^Department of Nursing, University of Girona, Girona, Spain; ^7^Independent Researcher, Barcelona, Spain; ^8^Fundació Institut Universitari per la Recerca a l'Atenció Primària de Salut Jordi Gol i Gurina (IDIAPJGol), Barcelona, Spain; ^9^Universitat Autònoma de Barcelona (Cerdanyola del Vallès), Bellaterra, Spain

**Keywords:** caregivers, informal caregiving, Covid-19 pandemic, salutogenesis, community engagement

## Abstract

**Background:**

Informal caregivers are essential figures that deal with the effects of dependence in the elderly. However, they suffer from poorer health-related quality of life, particularly regarding mental health. Social support is crucial, but this was suspended or dramatically reduced during the Covid-19 pandemic. Salutogenesis theory explores the contributing factors for the promotion and maintenance of health. Considering all these, we offered caregivers the opportunity to join a participatory project aimed at creating communication spaces where they could share experiences, think together about potential solutions, and explore which salutogenic actions they used in their daily basis and how they had changed during Covid-19 restrictions.

**Methods:**

We used a qualitative methodology with a socio-constructivist and phenomenological approach and purposive sampling. We organized two focus groups consisting of online semi-structured discussions with seven participants in total. Conversations were videotaped and transcribed and we conducted content thematic analyses using the NVivo software.

**Results:**

Caregiving in our setting are primarily women with high levels of education that do not always feel comfortable with this load because it interferes with their personal and professional lives. The pandemic increased caregivers feelings of loneliness, resignation, and burden, directly affecting their mental health. Furthermore, the disappearance of prevention programs and the difficulties to access healthcare services produced negative consequences on the already fragile elderly and their family caregivers.

**Conclusion:**

The pandemic and its restrictions exacerbated the problematics affecting informal caregivers. Although these people are aware of their situation and have valued knowledge of how to improve their health, they cannot always put it into practice. We call policymakers to reframe interventions aimed at caregivers by introducing the voice of the community in the planning and to rethink the management of vulnerable people and their carers in other potential health crises.

## 1 Introduction

Spain is at the top 10 of the Organization for Economic Co-operation and Development (OECD) ranking regarding the frequency and intensity of informal care (1). Informal caregivers (IC hereafter) are people who take care of family or friends, usually without economical retribution. In 2018, 12.4% of the elderly residents of Barcelona required IC (2). Informal caregiving is shaped by the social context of each country and the polices that they stablish to support IC. Differences are observed in this regard because the perceived duty to care for relatives varies across countries. In family-based societies and more deprived countries, informal caregiving prevails professional care (3). Relevant gender differences have also been identified throughout the world. For instance, in Barcelona, women do not only double the number of IC compared to men, but they also tend to take care of non-close relatives (2). In addition, as informal caregiving is unpaid work, it can become a private and domestic task undervalued both socially and economically. Oftentimes, this leads female IC to accept precarious jobs with worse working conditions and/or part-time positions, which has an obvious impact on their careers and quality of life (1).

Many studies have shown that IC suffer from poorer health-related quality of life than people of similar age, gender, ethnicity, and level of social deprivation (4, 5). Gonzalez-de Paz et al. (5) observed that IC are more likely to have received diagnoses of depression and anxiety and tend to report worse psychological well-being overall. Moreover, IC declare having a worse experience with healthcare services concerning the access and use of community care compared to other populations, which results in less support and more barriers and burdens (6). For instance, as it has been described in the study of Martin et al. (7), IC are often relied upon to ensure that patients adhere safely to treatment and to monitor any untoward side effects. However, IC often believe that healthcare professionals neglect them when it is time to evaluate different treatments options even if new disease management alternatives end up in new additional burdens for them. Furthermore, their role in the decision-making process can be confusing when the person who cares presents any kind of cognitive impairment such as chronic or temporary condition, as it happened with the covid-19 vaccinations (8).

Salutogenesis is the human health approach that examines the contributing factors to the promotion and maintenance of health (9). It is based on two fundamental concepts: Generalized Resistance Resources (GRR) and the Sense of Coherence (SOC). GRR are resources found within an individual or in his/her environment that can be used to counteract the stressors of everyday life and construct coherent live experiences (10). The SOC is defined as “a global orientation that expresses the extent to which one has a pervasive, enduring through dynamic feeling of confidence: (a) that the stimuli deriving from one’s internal and external environments in the course of living are structured, predictable, and explicable; (b) that the resources are available to one to meet the demands posed by these stimuli; and (c) that these demands are challenges worthy of investment and engagement” (9).

Findings suggest that the SOC could be an important determinant of IC’s well-being and may protect them from high levels of psychological distress and caregiver burden (10, 11). Moreover, studies have shown that the SOC might be a particular protective factor against subjective hardship, anxiety, and depression in IC of elderly dependent relatives (12). In fact, a significant reverse association was found between the burden of care and the SOC’s meaningfulness factor (13). There are also documented experiences of the use of the salutogenesis concept to improve IC’s wellbeing. For example, Wennerberg et al. (14) found a positive correlation between GRR and IC’s SOC applying guided interviews with a salutogenic approach. The interviews seemed to provide a reflective experience, mostly positive, empowering and enlightening, due to the focus on health improvement and the positive aspects of a situation that is usually described in negative terms. Similarly, Agulló-Cantos et al. (15) conducted 45 interviews to IC with a salutogenic perspective and observed that, even though these people are exposed to a source of stress, caregiving might act as a GRR since they can obtain positive experiences from being IC which can positively influence their health.

On a related note, literature has shown that highly participatory projects contribute to an enhanced understanding of the community assets and needs and contributes to strengthen empowerment and agency (16). Participatory Research Actions (PAR) aim to rethink our interventions by introducing the voice of the targeted population in the planning process. Through such a methodology, we ensure that problems are contextualized and interventions are tailored for the community in study (17). PAR have been reported to: (a) produce sustained collaborative efforts toward health improvement, (b) generate spin-off projects, and (c) achieve systemic transformations (16). Overall, shaping outcomes together and using participation techniques can encourage the use of resources to respond to internal and external stimuli (17) and provide purpose and a sense of belonging. There are documented experiences of PAR projects with IC that showed that their insights and suggestions enabled institutions to shape effective and successful interventions for them and their relatives (18).

For this reason, we created a salutogenic and participatory project for IC in three different health care centers of Barcelona: the INTerACT Project (INTroducing bidirectionality to the community: a salutogenic participatory Research ACTion in caregivers). The project had the objective to build bidirectional relationships with healthcare professionals by enabling communication spaces where IC could identify their problems and think together about potential solutions to improve their health and wellbeing. The project was started in 2019 but, shortly after, Covid-19 stroke. Social isolation measures hit the hardest in the most vulnerable, IC among them. In front of this situation, the IC involved with INTerACT reached out to the healthcare promotion professionals to find a way to keep sharing experiences.

During 2020, academics showed their concerns about the potential mental health repercussions derived from the pandemic restrictions on vulnerable people, such as the IC community, calling researchers and funding bodies to focus their actions on them (19). Additionally, Greenberg et al.’s (20) review, highlighted the positive impact of coping strategies for IC during this critical period such as exercise, keeping contact with friends and relatives through social media, or sticking to daily routines. Nonetheless, we also found studies in the literature which stated that the effects of the suspension or dramatic reduction of support and care systems were gaged in IC, showing a notable increase of their burden, loneliness, and depressive symptomatology (21–23). All these papers collected data from online surveys or telephonic interviews. However, we found no participatory and salutogenic experiences tackling this issue in the literature.

Therefore, as the initially-divised face-to-face meetings were no longer a safe option given the particular circumstances of IC and their dependents, we redefined the methodology to enable the project to continue remotely. INTerACT was transformed then into INTerREDACT, where the added *RED* stands for ‘network’ in Spanish. This paper reports thus a specific participatory action initiated by IC in collaboration with healthcare professionals and aims to explore two main issues. Firstly, how these people lived the social isolation during the Covid-19 lockdown, focusing on which differences they experienced and how they felt providing care to their relatives. Secondly, we wanted to explore which salutogenic actions IC adopted to overcome this situation, asking for specific GRR that they used during this period.

## 2 Materials and methods

### 2.1 Study design

We defined a qualitative study adopting a phenomenological and socio-constructivist approach. We wanted to focus the study on the social phenomena through IC’s life stories by exploring the meaning and common characteristics of their daily life experiences. Likewise, we analyzed the data by contextualizing and understanding their narratives within their social context (24).

### 2.2 Target community, sampling, and recruitment

We identified participants for the INTerACT project using purposive sampling. Social workers from the three health centers of Barcelona provided us with an initial list of IC that had already participated in caregiving training courses and had showed high rates of engagement during those activities. Then, we completed our group of participants via snowball recruitment. That is, by asking the initial selected participants if they knew anyone else with similar views or situations that could also be interested in taking part in the research (25). IC joined the training courses if they commited to a minimum of 80% of the training and did not meet any of the following exclusion criteria: suffering from a non-stable or non-treated severe mental disorder, consuming any addictive substances, having any cognitive or relevant sensorial impairments, or were taking part in other IC training groups in a different center. All participants included were contacted by phone and underwent a personal interview in which we confirmed that they complied with the aforementioned criteria and we asked for confirmation of agreement on the objectives and design of the study. In the recruitment interview we also explored and registered their caring situation and gathered demographic data.

During the Covid-19 lockdown, we kept contact with the participants by phone. As explained above, when they asked to continue with the social interactions we introduced the digital aspect and INTerREDACT was born. For this specific subproject, we also used purposive sampling. In this case, we selected participants from the INTerACT pool that shared similar social and caregiving literacy and we took into account previous interactions between them reinforcing already-established relationships. Since this was a participatory study, the INTerREACT participants agreed on focusing the research on their perspectives as IC during the lockdown. They were also able to modify the scripts of the focus groups and, later on, share their views on the analysis and results if they deemed necessary.

### 2.3 Data collection

We organized two focus groups consisting of on-line semi-structured discussions. This methodology has been widely used in qualitative research and aims to explore a specific set of issues. Moderators often initiate the discussion by asking broad questions about the topic of interest and then they advanced to the focal issues. Although participants individually answer the facilitators’ questions, they are encouraged to talk and interact with each other. This technique is built on the notion that the group interaction promotes respondents to explore and clarify individual and shared perspectives (24, 26).

We created a topic guide that included two main items and several sub-items (see Table 1). This guide worked as a check-list of the issues to cover during the sessions, but the focus group methodology framework enabled us to explore other topics that emerged from the participants’ interactions. The first item was an exploratory view into their experience during the lockdown. The second had the aim to observe their salutogenic actions during that time. We created an additional moderators’ guide with detailed prompts and which contained an approximate schedule for each topic to ensure that the semi-structured discussions were successful. The content of the topic guide was presented to the participants and they were able to propose changes if they considered necessary. The two researchers of the INTerREDACT project acted as moderators. In each session, we outlined the objectives and the functioning of the focus group and then we started the discussion. Moderators registered relevant notes about the issues that were discussed and the social interaction of participants during the focus group. We videotaped the focus groups and the audio was transcribed afterwards.

**Table 1:** Topic guide.

Caring experiences during the lockdown
How did you manage with the remote working?
Which differences have you observed regarding the medical attention?
How did you felt with these differences?
Experiences that helped you to get care of yourselves and improve your wellbeing during this period
Which actions had made you feel good mentally and physically?
Which actions that you had not made do you think it would have help you to feel better?
Have you used digital resources during the lockdown?
What rewards you from this situation?

### 2.4 Data analyses

The qualitative data were analyzed by two different researchers using content analysis. Krippendorff (27) defined content analysis as “a research technique for making replicable and valid inferences from texts (or other meaningful matter) to the contexts of their use.” The process followed in conducting qualitative content analysis is composed of four stages: decontextualization, recontextualization, categorization, and compilation (28). To increase the validity of all the results, the topics were discussed and clarified until a consensus was reached (29).

### 2.5 Ethics approval and informed consent

The Ethical Committee (EC) of Hospital Clínic granted the approval for this study through the submission of an amendment of the INTerACT protocol (previously approved by the same EC) and registered with the reference number HCB/2020/0396. The EC ensured that the study followed the ethical principles laid down by the Helsinki Declaration (30) and all applicable legal laws.

After the first in-person INTerACT interview, we conducted phone interviews explaining INTerREDACT and then sent the documentation to sign the specific informed consent ensuring participants’ anonymity, data confidentiality, and the possibility of withdrawal from the project. Another consent for voice and image recording was used for the sessions to be recorded and verbatim transcribed. Participants were informed that the results could be shared for research purposes.

## 3 Results

### 3.1 Participants

A total of seven people participated in the two focus groups; one with four participants and the other with three (see Table 2). All of them were women over 50 years old (range: 50–75). They came from the three different health care centers from the same Barcelona city district. Six of them had higher education. We have used pseudonyms to refer to the different participants throughout the document.

**Table 2:** Participants’ characteristics.

Participants	Gender	Age	Employment status	Civil status	Education level
Monica	Female	66	Unemployed	Single	Higher
Maria	Female	75	Retired	Married	Secondary
Evelina	Female	55	Unemployed	Separated	Higher
Sara	Female	58	Active	Separated	Higher
Henar	Female	57	Active	Married	Higher
Helena	Female	68	Retired	Single	Higher
Amelia	Female	54	Active	Divorced	Higher

All participants took care of a relative with some degree of cognitive impairment. Monica, Evelina, Sara, Amelia, and Helena took care and lived with their mothers. Henar took care of both of her parents, who lived outside the family household. Maria was an IC for her husband. Evelina, Sara, Henar, and Amelia had also dependent children. Besides her mother, Helena took care of two brothers with mental health diseases who lived outside the family household.

### 3.2 Group dynamics

**Figure 1:**
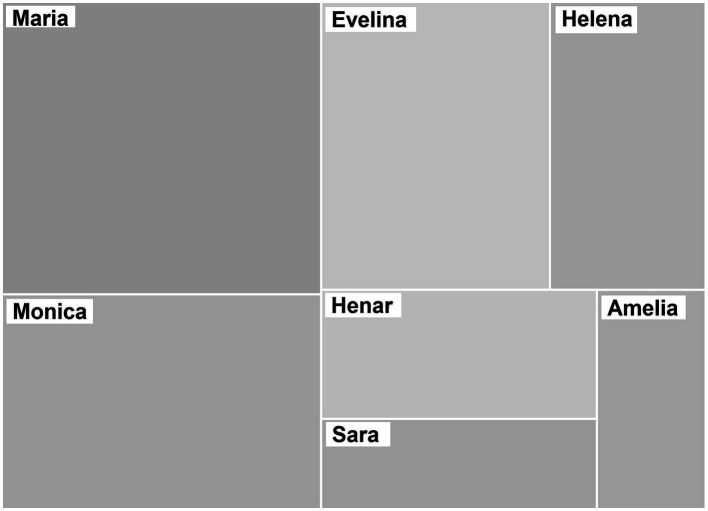
Distribution of IC’s participation during the focus groups. The sizes of the rectangles correspond to the frequency of participation of each IC.

We found some differences in participation time among IC (see Figure 1). Maria and Monica were the most active and Amelia and Sara the least. However, we can observe that all of them contributed to the focus groups and expressed their opinion.

### 3.3 Discourse analyses

The main issues covered during both focus groups were: (a) aspects related to the caregiver’s context, (b) experiences of informal caregiving during the lockdown and the pandemic situation, and (c) salutogenic resources that IC usually employ to overcome their situation and those that they particularly used during the lockdown and the pandemic situation.

#### 3.3.1 Informal caregivers’ context

During the focus groups, IC described their context focusing mainly on their feelings about caring, but also how this situation affected their mental health and their caring perspectives, as well as which socioeconomic factors were involved.

**Figure 2:**
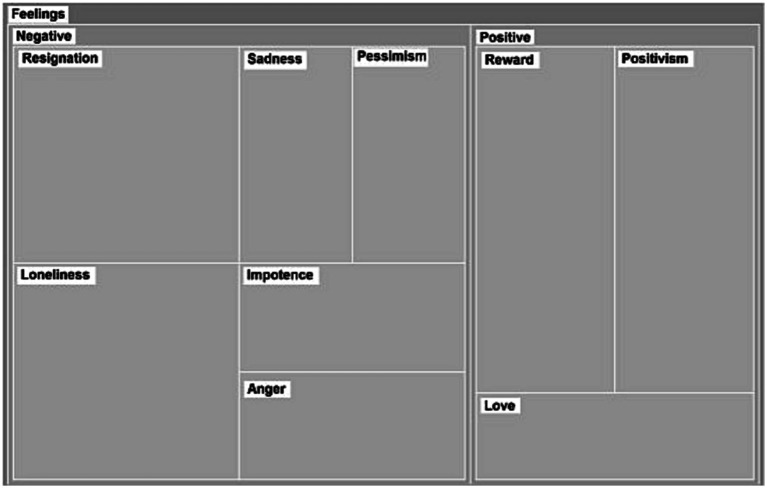
Categories and subcategories of expressed feelings. The sizes of the rectangles correspond to frequency of appearance of each topic.

Loneliness and resignation were the dominant feelings in our conversations (see Figure 2). However, while resignation was indirectly expressed, loneliness was verbally specified during the conversations. Moreover, IC expressed that they felt they had to carry the weight of caregiving by themselves, even when there were other family members. This feeling was increased during the pandemic because they considered that healthcare professionals were not present the way they expected them to be.

*“… lonely, lonely, I mean I know I am with other people, lonely in the sense that I do not have the knowledge, I mean, that you do not know…* “*(Henar)*.

*“… I felt very isolated, very lonely, without doctors, with no one that could come to, to help…” (Evelina)*.

Resignation was widely identified in the transcripts, as the participants expressed discomfort in their current caregiving situation. They declared how they had to renounce to aspects of their life in order to provide assistance to their relatives. Notwithstanding, they usually found balanced feelings acknowledging also the associated rewards of caregiving and the love received by their relatives.

*“… I feel rewarded for what I am doing, but I feel also bitter in my life, I cannot deny…” (Monica)*.

When talking about mental health issues and caregiving, they expressed the psychological consequences of overcoming daily problems related with their relatives’ diseases. The most common symptom among participants was burden, but they also highlighted the loss of personal space and anxiety-related feelings.

*“… I was putting up with the situation, but I was really at my limit. My limit of what I can endure. Every day, I was going to sleep asking for help, help, and that, at least, I could wake up. It was as if I woke up with a different mood and I could put up with the whole day and again another night…” (Evelina)*.

However, it was interesting to observe the empowerment one specific participant obtained from her condition.

*“… I grew stronger. I am stronger in the sense that I had to work internally with my emotions. I had to have more patience…. I think that this has made us stronger and that we got to know more about ourselves…” (Monica)*.

Most of the participants felt that home-caring was an option they wanted to choose for their elderly, and they criticized people choosing other forms of caring. But one particular IC, Sara, said she regarded home-caring as a natural process in life and not an option, highlighting the fact that she is Colombian and, in her country, this conception is normalized. In addition, as shown in other studies, IC criticize the expenses of formal care and the difficulties in getting benefits from the government due to bureaucracy (3).

Amelia also introduced the gender perspective of caring, stressing how women usually adopt the caregiver role while carrying out their professional careers with little help of other family members or with no formal support available.

*“… as women, we need to overwork. I am a consultant and I work during nights losing sleeping hours. I have to look for someone to watch out my mother, well, television watches her out and sometimes my son…” (Amelia)*.

There were also participants such as Evelina or Henar who had to stop working in order to take care of their family.

*“… I did it, what I am doing is right, but when I look backwards, and I see what I had to renounce to, I was an economist, I had a profession, I had responsibilities, I enjoyed my life… I had to renounce… I have become a housewife and caregiver, but this wasn’t on my list of things to do in life…” (Henar)*.

#### 3.3.2 Experience of informal caregiving during the lockdown and the pandemic situation

**Figure 3:**
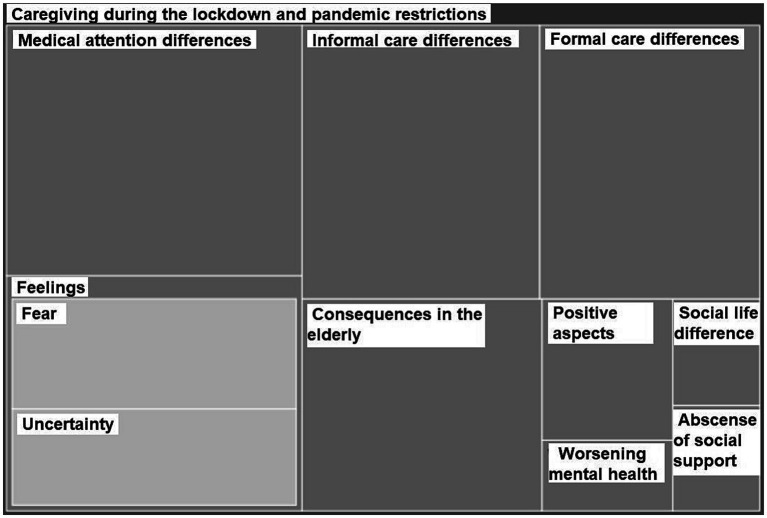
Subcategories of caregiving during the lockdown and the pandemic restrictions. The sizes of the rectangles correspond to frequency of appearance of each topic.

During the pandemic, IC were initially home-locked with their relatives for 3 months and an extended period of social restrictions, which kept interfering in their caring situation, followed for several months. This situation changed their context, particularly in the way the medical and the formal care was provided, and also in the support they received from their social network, which was mostly already scarce. Figure 3 shows the most popular topics related to this area.

The participants were not willing to use hospital services because there were strict restrictions concerning visits and they feared that the elderly would be alone with high odds of a fatal situation occurring because of their fragility or them being more disorientated.

*“… if you want, you can visit (my mother) here but not at the hospital, because she is completely vulnerable… I understand that they are doing it for her own good, but my mother already has lived her life. There is no need, if it’s her time I would rather be with her… I do not know if I will be able to do it, but I do prefer to be with her, holding her hand…” (Helena)*.

*“… I would not take my mother to the hospital, because I knew that if I took her to a hospital, she would not get back… “(Evelina)*.

Furthermore, most of the participants felt neglected by the medical care authorities and had issues with remote medical attention.

*“… I did not receive any calls from the health center to check on her, not even once…” (Amelia)*.

*“… something that worried me a lot during the lockdown were the medical appointments, because the doctor called us: How are your parents doing? I did not know, how could I tell them, I really did not know if they had something serious, I cannot know this, I am an economist not a doctor…” (Henar)*.

Day care centers closed and short-stay residence programs were canceled. Moreover, some of them opted out from the help that they were getting from formal caregivers to reduce the number of contacts and avoid chances of Covid-19 infection. Later on, during the restrictions period and with the reopening of the day centers, many of them were not willing to re-enroll them because they still feared that their elderly could get infected.

Therefore, due to the pandemic restrictions, most of the IC reported that they were assuming all the caring load in order to reduce social contacts. These changes in the care support interfered with the IC’s work and other personal tasks, increasing the risk of burden. They also felt that the situation worsened the cognitive capacities of the elderly.

*“… my sister sometimes comes once or twice a week, but since she lives in Tarragona she cannot come because of the restrictions…” (Monica)*.

*“… My father has lost a lot with the pandemic…. he used to read the newspapers, did additions, write… now he does not even bring his planner. He used to talk with everyone…” (Henar)*.

During the focus groups, participants got emotional because they mentioned the hard times they suffered fearing about the safety of their relatives and not being able to control the situation because of the general uncertainty of the moment.

Nevertheless, it was interesting to observe the resilience of some of the participants finding also positive aspects among this situation. They especially valued the time spent with their relatives:

*“… for me, it was beneficial because I am always working… and it was a moment of being back all together…” (Sara)*.

#### 3.3.3 Salutogenic resources

**Figure 4:**
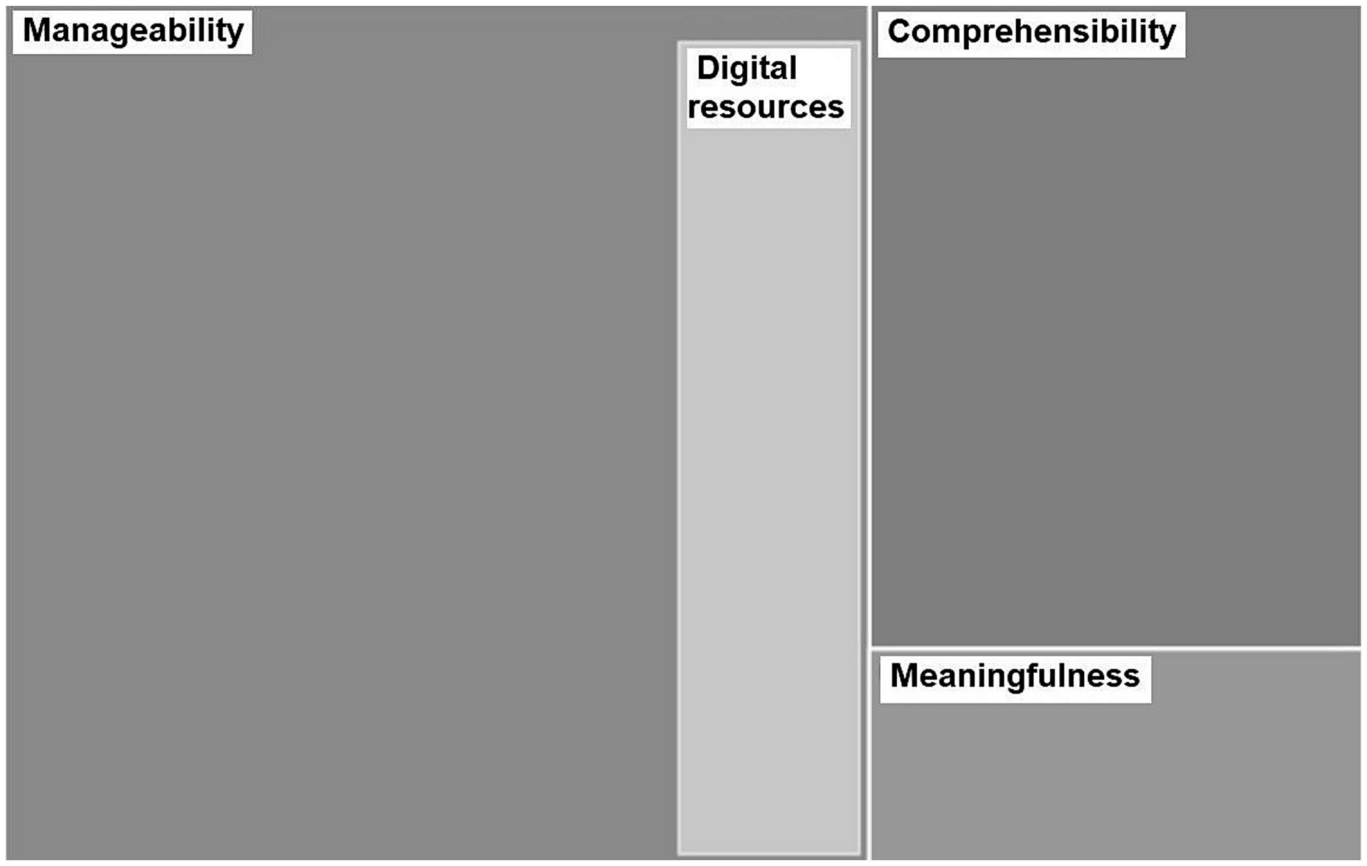
Subcategories of salutogenic dimensions. The sizes of the rectangles correspond to frequency of appearance of each topic.

Discourse was categorized following salutogenic principles: comprehensibility, manageability, and meaningfulness. We paid special attention to the examination of the possible role of the digital resources during the pandemic, included in the manageability sphere (Figure 4).

The comprehensibility dimension in the salutogenic theory expresses that the stimuli deriving from one’s internal and external environments in the course of living are structured, predictable, and explicable. During the focus group, we observed that IC had gone through an introspective process to get know themselves better and learn about their needs and acceptance of their situation.

*“… I’ve learnt to downplay things to relativise things. To stop planning that much…. I planned too much, one day here another there, everything under control, everything must be arranged, I need to have everything alright. Now I take things slowly, I’ve learnt that I am able to calm down, because otherwise I cannot do anything but destroy myself. “(Sara)*.

*“… I am two hours alone, I am always going to sleep very late, but it is what I need, some time for me…” (Maria)*.

The manageability dimension means that the resources are available to one to meet the demands posed by these stimuli. IC showed us that they used plenty of resources that helped them to overcome their situation. Economic resources such as the benefits that the government provides for the elderly dependent in Spain, the use of their social network, or sport practice. Also, cultural resources like reading, music, photography, meditation, or mindfulness.

*“… during the pandemic I worked out by going upstairs and downstairs, I moved. I also sunbathed with my mom in the balcony… These things helped me to take good care of my body… These things help me…” (Helena)*.

Broadly speaking, taking time for themselves, which allowed them to perform these activities, was reported as a widely used and valued tool to keep them healthy. Notwithstanding, time for themselves was claimed to be the hardest to achieve, as Monica clearly exemplifies in the following verbatim:

*“… I see my sister as heavenly help. When she comes, she cooks, she is the caregiver. She makes all this, but I say: there are 365 days and you help 2 of them, that means 363 for me…” (Monica)*.

Finally, with the pandemic, digital resources became a primordial connection with society and our IC took advantage of them. Video conferences with friends, YouTube tutorials for practicing sport, or inspirational speeches were some of the on-line resources they mentioned. During the lockdown, Evelina also found comfort in an Instagram account of a famous Spanish actor who decided to confine with her grandmother and showed their daily life together.

Lastly, the meaningfulness dimension introduces the concept that the demands posed by the stimuli deriving from one’s internal and external environments are challenges worthy of investment and engagement. Broadly, IC agreed that the core reason that made them take the decision to become IC was because they thought it is the right thing to do and they receive the love from their relatives.

*“…When I see my mother so well, and well-cared for, I swear I experience great joy, when she kisses me and tells me how beautiful I am I know she is all right, that I’ve done what I had to do, and I will do it again…” (Henar)*.

## 4 Discussion

In our setting, informal caregivers (IC) were primarily women with high levels of education that do not always feel comfortable with this load because it interferes in their personal and professional lives. On top of this structural hardship, the Covid-19 pandemic and the consequent social restriction policies imposed worsened their situation. During that period, they experienced increased feelings of loneliness, resignation, and burden, directly affecting their mental health. Furthermore, the disappearance of prevention programs and the difficulties to access healthcare services, produced negative consequences on their fragile elderly dependents and themselves. The IC studied here showed good comprehension of their obstacles and had the knowledge to improve their health, but we observed that they were not always able to put their own coping strategies into practice. We believe that the focus group technique allowed us and our participants to better and more profoundly understand their problems and helped to build community-engagement among caregiving peers.

When we analyzed their dialogs, the most prominent observation was that participants believed that taking care of their relatives is a duty that they have to accomplish, but which causes contradictory feelings. On the one hand, they valued being able to provide care in their home settings and they were able to get positive experiences from it, such as love and reward. However, on the other hand, this situation interfered with their professional careers and social life and, consequently, raised frustrating feelings. As it happened to some participants of our study, Pickard et al. (31) evidenced that many IC move to part-time paid employment or just leave their jobs, with direct consequences not only to themselves and their families but also to the society as a whole. Our participants’ professional interferences cause social exclusion, which results in the inability to participate in ordinary relationships and activities available to the majority of people in the society (32).

Another important aspect that surfaced during the study was the issue of gender. Jimenez and Moya (33) conducted a qualitative study about women’s naturalization of the caregiver role. Their findings suggested that women usually hold a moral and emotional duty to care for the family but, at the same time, they desire their own personal development. Similarly, the 2018’s Barcelona Women Caregivers Report (34) concluded that this moral sense of duty usually makes women to do this work alone, often having troubles finding consistent help from other family members. Our participants repeatedly reported this struggle and they expressed strong feelings of loneliness associated with it. These findings are congruent with other studies in which it is shown that women tended to assume more caregiving tasks than men during the pandemic and also expressed more burden than men (35, 36).

Relatedly, before the pandemic, our IC usually benefitted from other formal aid provided by the government or their own private resources, which reduced their daily caring load. Some of them also had available informal aid like family members or friends that could occasionally help them out with their elderly or give them emotional support. In the Spanish context, formal aid is provided by the dependence law (37), which provides relatives of dependent people with family workers’ hours to attend the dependent person or a monthly payment to help families with the expenses of the caregiving task. There are other formal and local programs aimed mainly at improving the functionality of dependent people and reduce the load of caregivers, such as day-care centers, which vary across city districts or towns. However, when Covid-19 stroke, they lost this support net which IC heavily depend on (e.g., closure of day centers or reduction or abolition of formal caregiving solutions to minimize social contact). Therefore, their basal feelings of loneliness and burden raised, impairing their mental health status. These findings agree with the literature found in other countries (38, 39). For instance, in the UK, Gallagher and Wetherell (22) analyzed data from a national survey during the first year of the pandemic and they observed that higher levels of loneliness increased the risk of depression symptoms almost four-fold in IC.

Moreover, during this period, participants particularly highlighted the troubles in getting medical attention for their elderly. They narrated different experiences in which their relatives got medical conditions that required from the evaluation of healthcare providers but community healthcare centers had limited their access, prioritizing remote channels to respond to health demands. IC felt neglected and helpless without face-to-face assistance. IC did not felt comfortable with the remote support of healthcare workers, as they believed that they were not able to properly explain the health problem of their relative and they feared they might miss something relevant. The problematics of access to healthcare services and its consequences during the pandemic scenario, particularly for chronic patients, have been also described in other Spanish regions (40). However, regarding the emerging technological solutions where caring is involved, reports from caregivers from other countries contrast those from Spain, as these technologies have been considered to enable rather than hinder the attention of people with dementia (41).

Participants were also sometimes reluctant to seek help in the hospital setting since companions were restricted during the hospitalization, and they feared that their elderly could get a fatal Covid-19 infection there. In the Canadian study of Hindmarch et al. (42) was proved that visitor restrictions during the pandemic produced negative outcomes to IC, including social isolation, strain, and reduced quality of life. Similarly, other studies (21, 38, 43) have pointed out that one of the consequences of the lockdown and social restrictions in the elderly affected with dementia was the worsening of their cognitive impairment and behavior.

With respect to the participants’ salutogenic agency, we could assess that, although they were not always able to apply them, overall, they were aware of the elements that help them moving toward a healthy and good quality of life. Such knowledge was shown to have a protective effect on the psychological state of IC during the pandemic (44). The use of on-line resources had also a positive impact on our participants, incrementing their resilience mechanisms to overcome that particular scenario. This correlates with the study of Yoon et al. (45), which examined topics and feelings expressed by IC on Twitter and concluded that on-line social media have the potential to be a platform to promote positive coping strategies and resilience.

Compared to other investigations, the present study did not only aim to collect data, but also to build a social network among IC that will be later involved in a larger project to reframe their relationships with healthcare providers. A participatory action research intervention that helped to think together about IC’s needs and possible solutions in their context. We found different qualitative studies in the literature involving IC and the Covid-19 pandemic, mostly individual surveys (21, 22, 42–44, 46–48). However, the focus groups here had the purpose of giving voice to participants to express themselves comfortably, creating a trust space between equals. We believe that this structured methodology, which additionally followed the COREQ checklist (26), enriched the discourses and facilitated the creation of social connections. We also think that while many studies have focused on gaging the depressive, anxiety, and burden symptoms that IC suffered during the pandemic, the qualitative approach used in this research facilitated exploring the triggers of these feelings. Furthermore, the salutogenic perspective that we introduced promoted participants’ recognition of their own coping strategies to overcome the daily obstacles that they faced and those derived from the pandemic situation.

### 4.1 Limitations of the study

On the one hand, while the sample of the present study is small, we believe that the women studied (above their 50s, some of them already retired and with a background of high levels of education) are representative of the upper-middle socioeconomic context of the Barcelona city district where the project was set and can be extrapolated to the target population with a certain degree of confidence. On the other hand, we acknowledge that the use of a purposive sample with highly experienced IC (both in terms of caregiving itself and informal caregiving group training) could somewhat hinder its projection. However, we were interested in that the data gathered was knowledgeable, as this intervention is the first of a series of a larger project involving other IC from the same city district and has the purpose to help us build solutions to apply in our primary care setting. On a different note, the fact that the focus groups needed to be conducted on-line had the advantage that we could connect despite social restrictions and it was more practical for IC given that their schedules were already limited due to their caring obligations. Despite this, videoconferences may interfere with fluent communication because of connection problems and they can hinder natural human interactions, as well as inhibit some non-verbal communication that is also relevant for qualitative studies. Therefore, although digitalization tools in general have proven to mitigate the burden of care for caregivers (49), they may have damaged the quality of social interaction that we pursued in this intervention.

### 4.2 Implications for clinical practice

The pandemic crisis was an unfortunate opportunity to expose the vulnerability of the care system of our society, and it has been useful to rise awareness about how institutions may respond to the most fragile. The salutogenic perspective and qualitative methods of the present study allow not only to deeply understand the problematic of this population, but also to focus on their coping strategies. This data can be useful to build new intervention programs adjusted to the IC’s daily needs and in potential future health crisis. For this purpose, we believe that we need new participatory action research focused on understing IC’s social phenomena to build, together with them, eventual interventions directed to their community.

### 4.3 Conclusion

In our setting, informal caregiving is a feminized population who expresses feelings of discomfort with its caregiving activity, as it hinders women’s personal and professional development. Moreover, they referred feelings of loneliness, resignation, and burden that affect their mental health. Social restriction policies during the pandemic had a direct effect on this group, increasing their social isolation. The absence of prevention programs for vulnerable people and the barriers to access healthcare services during this period, also produced negative consequences on the fragile elderly and their family caregivers. Informal caregivers are aware of their situation and have valued knowledge of how to improve their health, but oftentimes cannot apply it due to their intrinsic circumstances.

In order to improve the quality and the safety of the services aimed at caregivers, we call policymakers to reframe interventions aimed to them by introducing the voice of the community in the planning, and to rethink the management of vulnerable people and their carers for other potential health crisis. Based on the findings of this study, we suggest that institutions should focus on three key points:

1. Reducing the gender gap observed in IC by improving the reconciliation of informal care and paid work, by increasing the formal aid targeted to reduce the caregivers’ load, and by incentivizing women caregivers’ networks to enhance their resilience and reduce and share their burden.
2. Rethinking the management of formal aid provided for the dependent population during a pandemic by finding new formulas to keep this population active and, at the same time, by aiding caregivers with their caring tasks.
3. Healthcare institutions should improve telemedicine and communications targeted at vulnerable people and transform them into a more satisfying experience for families and patients.

## Data availability statement

The raw data supporting the conclusions of this article will be made available by the authors, without undue reservation. Requests to access these datasets should be directed to the corresponding author.

## Ethics statement

The studies involving humans were approved by Ethical Committee (EC) of Hospital Clínic. The studies were conducted in accordance with the local legislation and institutional requirements. The participants provided their written informed consent to participate in this study. Written informed consent was obtained from the individual(s) for the publication of any potentially identifiable images or data included in this article.

## Author contributions

OM-C and LT-P: study design, recruiting, interviewing, data gathering, transcription, data analysis, and writing manuscript. AM-C: writing, reviewing, and editing. GR-G, AB, and DJ-C: reviewing and editing, visualization, and supervision. All authors have read and agreed to the published version of the manuscript.
